# Cleaning and Durability of Upper Extremity Orthotics: A Patient's Perspective

**DOI:** 10.7759/cureus.66794

**Published:** 2024-08-13

**Authors:** Tiffany N Bridges, Quincy T Cheesman, Matthew H Meade, Adam S Kohring, Joseph McCahon, Britani Walsh, Amir Kachooei, Pedro K Beredjiklian, Michael Rivlin

**Affiliations:** 1 Orthopaedic Surgery, Jefferson Health New Jersey, Stratford, USA; 2 Hand Surgery, Rothman Orthopaedic Institute, Philadelphia, USA; 3 Hand Surgery, Rothman Orthopaedic Institute, Orlando, USA

**Keywords:** thermoplastic material, orthotic, instructions, fiberglass, cast immobilization

## Abstract

Introduction

We aimed to evaluate orthotic hygiene, preference for immobilization material, and frequency of unplanned orthotic adjustments and replacements.

Methods

All patients with fiberglass casts, thermoplastic splints, or prefabricated braces who presented at a large private academic institution between January 2020 and July 2023 were provided an 11-item survey assessing the length of immobilization, frequency of orthotic changes, orthotic hygiene, preference of immobilization, and whether patients recall instructions regarding orthotic care.

Results

A total of 385 surveys were collected, consisting of 96 (24.9%) casts, 202 (52.5%) thermoplastic splints, and 87 (22.6%) prefabricated braces. Patients were most frequently immobilized for two to six weeks. Of those, 106 (27.5%) patients required an unplanned adjustment or replacement. Almost half (182 patients, 47.3%) attempted to clean their orthotics, which was significantly greater among thermoplastic splints. A total of 229 (59.5%) respondents reported either not receiving or were unsure if they received instruction on proper orthotic hygiene.

Conclusion

Orthotic care and hygiene instructions are often overlooked or not retained by patients. Nearly one-third of patients required an unplanned adjustment or replacement, which was most frequent with thermoplastic orthotics.

## Introduction

In orthopedics, the utilization of splint and cast orthotics is instrumental in facilitating the recovery process following a fracture. They serve as an integral component in stabilizing fractures, which often follow either a reduction or surgical intervention [1, 2]. Maintaining the cleanliness of a splint or cast is of paramount concern, as it is key to a successful and comfortable recovery. A clean splint or cast safeguards its structural integrity while also preventing infection. Additionally, a cosmetic, non-odorous, appearing splint or cast fosters a better experience for patients [3].

The American Academy of Orthopaedic Surgeons recommends maintaining a dry splint or cast while preventing dirt from entering the immobilization device. However, these recommendations are general in nature and lack an emphasis on the overall cleanliness of a splint or cast [4]. Anecdotally, there is a lack of consistency regarding patient education on the maintenance of splints or casts. It is unknown whether patients understand how to maintain the cleanliness and care of their splint or cast. The purpose of this study was to evaluate how patients maintained the cleanliness of their orthotics, their preference for immobilization, and how often adjustment or replacement was required. Knowledge of this information will allow providers to better educate patients.

## Materials and methods

Following institutional review board approval and waiver of informed consent, a prospective survey study was performed for patients with wrist or hand orthotics at a single institution from January 2020 to July 2023. All patients with fiberglass, thermoplastic, or prefabricated orthotics were provided an 11-item survey assessing length of immobilization, frequency of unplanned orthotic replacements, orthotic hygiene, including cleaning and drying methods, and preference for a waterproof orthotic. Patients were also asked if they recalled receiving instructions on orthotic hygiene.

Survey responses were collected once throughout their immobilization period. Only surveys completed in their entirety were included in the study. Patients with three-dimensional printed casts were excluded from the study. If the patient was under the age of 18, then responses were collected by the patient’s parent or legal guardian. Patients were divided into three cohorts based on their orthotics: fiberglass cast, thermoplastic splint, and prefabricated brace. Patients presenting with plaster splints, used to stabilize the initial reduction of a fracture, were included in the cast cohort if their plaster splint was removed on initial evaluation and subsequently wrapped with fiberglass material. All fiberglass casts and prefabricated braces were applied and fitted by certified orthotists. Additionally, all thermoplastic splints were fabricated and fitted by certified hand therapists at our institution. All orthotic recipients and their legal guardians, if applicable, were provided standardized written and verbal instructions, which were documented in the electronic medical record by the hand therapist or orthotist.

Statistical analyses were performed using Mann-Whitney U tests for continuous data and chi-square tests for categorical data. Statistical significance was defined as p < 0.05.

## Results

A total of 385 surveys were included in the study, consisting of 96 (24.9%) fiberglass casts, 202 (52.5%) thermoplastic splints, and 87 (22.6%) prefabricated braces (Table 1). On average, patients with thermoplastic splints were significantly older than those with casts and prefabricated braces (p < 0.001).

**Table 1:** Orthotic care and preference based on the material of immobilization x: times

	Cast	Thermoplastic splint	Prefabricated brace	P-value
	N = 96	N = 202	N = 87	
Age	18.5 (13.0; 60.0)	46.5 (24.0; 65.0)	41.0 (27.0; 63.0)	<0.001
Length of Immobilization				<0.001
<2 weeks	37 (38.5%)	18 (8.9%)	15 (17.2%)	
2 – 6 weeks	56 (58.3%)	126 (62.4%)	49 (56.3%)	
6 – 10 weeks	3 (3.1%)	45 (22.3%)	17 (19.5%)	
> 10 weeks	0	13 (6.4%)	6 (6.9%)	
Unplanned orthotic change	18 (18.8%)	65 (32.2%)	23 (26.4%)	0.051
Frequency of orthotic changes				0.253
1x	8 (44.4%)	41 (63.1%)	11 (47.8%)	
2x	8 (44.4%)	16 (24.6%)	6 (26.1%)	
>3x	2 (11.1%)	8 (12.3%)	6 (26.1%)	
Was the orthotic cleaned?	10 (10.4%)	146 (72.3%)	26 (29.9%)	<0.001
Frequency of cleaning				0.779
<1x/week	4 (40.0%)	46 (31.5%)	11 (42.3%)	
1-2x/week	4 (40.0%)	58 (39.7%)	10 (38.5%)	
>2x/week	2 (20.0%)	42 (28.8%)	5 (19.2%)	

A total of 182 (47.3%) patients reported cleaning their orthotics. Approximately three-fourths of those patients had thermoplastic splints (146 patients, 72.3%), which was significantly greater than those with fiberglass casts (10 patients, 10.4%) and prefabricated orthotics (26 patients, 29.9%) (p < 0.001). Patients most frequently reported cleaning their orthotic with a moist sponge (59 patients) or hand soap (59 patients). Among the 59 patients who reported using other methods, the most common cleaning modality was an alcohol or disinfecting wipe (51 patients), followed by washing machines (four patients), dish soap (two patients), and detergent (two patients). Pat drying with a cloth (95 patients) and air drying (91 patients) were the most frequently reported drying methods, followed by using a hair dryer (nine patients).

Orthotic replacements were reported among 106 (27.5%) survey respondents. Of those, 60 (56.6%) patients reported having their orthotic changed once, with the remainder reporting two (30 patients, 28.3%) or three or more (16 patients, 15.1%) replacements (Table 1). Orthotic changes were most frequent amongst thermoplastic splints (65 patients, 32.2%), followed by prefabricated braces (23 patients, 26.4%) and fiberglass casts (18 patients, 18.8%) (p = 0.051) (Table 1). More than half (61 patients, 57.5%) of the patients with unplanned orthotic changes cleaned their orthotic, which was significantly greater than patients who did not require an orthotic change (121 patients, 43.4%) (p = 0.018).

More than half of the patients either did not recall (166 patients, 43.1%) or were unsure (63 patients, 16.4%) if they received instruction regarding orthotic hygiene (Table 2). This was especially pronounced amongst patients with fiberglass casts (82 patients, 85.5%) and prefabricated braces (70 patients, 80.4%). Alternatively, there were a significantly greater number of patients with thermoplastic splints who recalled receiving orthotic care instructions (125 patients, 61.9%, p<0.001). Fewer patients with an unplanned orthotic change indicated that they received orthotic cleaning instructions; however, this did not reach significance (41 patients, 38.7% vs. 115 patients, 41.2%; p = 0.843).

**Table 2:** Orthotic care instruction and preferences based on unplanned changes and orthotic material

	Total	Unplanned orthotic changes			Orthotic material			
Variable		No change	Orthotic change	P-value	Fiberglass cast	Thermoplastic	Prefabricated	P-value
Did you receive the cleaning instructions? (%)				0.843				<0.001
No	166 (43.1)	120 (43.0)	46 (43.4)		71 (74.0)	38 (18.8)	57 (65.5)	
Yes	156 (40.5)	115 (41.2)	41 (38.7)		14 (14.6)	125 (61.9)	17 (19.5)	
Unsure	63 (16.4)	44 (15.8)	19 (17.9)		11 (11.5)	39 (19.3)	13 (14.9)	
If your cast was waterproof, would you have cleaned it? (%)				0.299				<0.001
No	57 (14.8)	46 (16.5)	11 (10.4)		28 (29.2)	13 (6.44)	16 (18.4)	
Yes	219 (56.9)	157 (56.3)	62 (58.5)		35 (36.5)	139 (68.8)	45 (51.7)	
Unsure	109 (28.3)	76 (27.2)	33 (31.1)		33 (34.4)	50 (24.8)	26 (29.9)	
Do you prefer a fully waterproof option? (%)				0.025				0.009
No	85 (22.1)	69 (24.7)	15 (15.1)		33 (34.4)	32 (15.8)	20 (23.0)	
Yes	167 (43.4)	110 (39.4)	57 (53.8)		35 (36.5)	97 (48.0)	35 (40.2)	
Unsure	133 (34.5)	100 (35.8)	33 (31.1)		28 (29.2)	73 (36.1)	32 (36.8)	

In general, 167 (43.4%) patients preferred a waterproof orthotic, which was significantly greater amongst thermoplastic splints (thermoplastic splint: 97 patients (48.0%), fiberglass cast: 35 patients (36.5%), prefabricated brace: 35 patients (40.2%), p = 0.009), and those who required an orthotic change (57 patients (53.8%) vs 110 patients (39.4%), p = 0.025) (Table 2). If provided a fully waterproof option, a significantly greater number of patients with thermoplastic splints reported that they would have cleaned it (thermoplastic splint: 139 patients (68.8%), prefabricated brace: 45 patients (51.7%), fiberglass cast: 35 patients (36.5%), p<0.001).

## Discussion

The current study demonstrated that almost a third of patients with a correctly fitted orthotic required at least one unplanned orthotic change during their course of immobilization. This was more frequent amongst those who cleaned their orthotics. Although appropriate for waterproof modalities, cleaning water-susceptible orthotics can compromise the integrity of the material and trap moisture. This can create an ideal environment for microbial proliferation. In fact, even in a dry environment, orthotics have been shown to harbor both normal skin flora and opportunistic pathogens [5].

Improper orthotic care can lead to skin irritation, breakdown, and infection [6-10]. To avoid such complications, damaged or non-waterproof orthotics that become wet are often removed to evaluate the skin and then subsequently reapplied. This often results in visits to the emergency department, especially in pediatric patients [1]. In a prospective study involving 1,135 casts, DiPaola et al. demonstrated that 5.3% of nonwaterproof casts required an unplanned change, which was most commonly due to wetness. This was even higher in a retrospective study by Sawyer et al., where 29% of casts were changed due to being wet [8].

Interim orthotic changes carry risks as well. Irrespective of material, orthotic changes can lead to loss of reduction and/or stability. Iatrogenic thermal and abrasive injury can also occur following the removal of fiberglass casts [11]. Additionally, unplanned orthotic changes may be associated with increased cost and resource utilization. Sawyer et al. demonstrated that 168 emergency room visits for cast-related issues over a five-year period imposed an additional charge of $126,374 on the healthcare system. Of those, 59% involved an orthotic change, while the remainder consisted of providing reassurance, bivalving, pain control, or removing the orthotic [8]. Removing and applying casts is time-consuming for the orthotist, and manufacturing and refitting thermoplastic and/or prefabricated orthotics carries a significant cost that may not be reimbursed for providers. Therefore, unplanned orthotic changes should be limited.

Orthotic changes were most frequent amongst thermoplastics, followed by prefabricated orthotics, however, this difference did not attain statistical significance. This may be due to a higher proportion of patients receiving thermoplastic splints in our population. Alternatively, thermoplastic and even prefabricated orthotics are susceptible to deformation and fatigue over time, which can contribute to unplanned replacements [12].

Despite providing routine verbal and written instructions regarding proper orthotic hygiene to all patients within our institution, 59.5% of respondents reported either not receiving or were unsure if they received instruction on proper orthotic hygiene. This was higher amongst patients who required an orthotic replacement. Patients with thermoplastic splints were more likely to report receiving cleaning instructions than those with prefabricated and fiberglass orthotics. This may be due to the one-on-one time spent with patients by the hand therapists versus the orthotist. However, further evaluation of this discrepancy is warranted.

Improved patient education may help reduce unplanned replacements and ultimately the financial burden experienced by patients and the healthcare system. However, both Sawyer et al. and DiPaola et al. reported noncompliance to proper orthotic hygiene amongst pediatric patients despite both verbal and written instruction [3, 8]. Newman et al. demonstrated similar rates of unplanned office visits due to cast noncompliance when comparing verbal instructions alone to verbal and written instructions [13]. Interestingly, when the written handout was revised to include a cartoon and condensed to only include instructions to avoid getting the cast wet, the number of unplanned office visits decreased but did not attain statistical significance. It is possible that alternative forms of education, such as video instruction, should be employed, as they have been shown to improve comprehension and compliance with perioperative instruction [14, 15]. However, further studies are required to assess its role in reducing unplanned orthotic changes.

Overall, 43.4% of the patients preferred a fully waterproof orthotic, while approximately one-third of patients were unsure. Prefabricated orthotics, although water-susceptible, present a unique situation in that they can accommodate temporary removal for hygiene if advised by the prescriber. Other alternatives in modern orthotics may lead to the adoption of more patient-friendly options, minimizing the need to revise them.

Based on our knowledge, this is the first study assessing patient-reported orthotic care and unplanned replacements. However, our study has limitations. First, patients completed the survey during their immobilization period, which may not have correlated with the end of their treatment. Therefore, it is possible that some patients underwent subsequent orthotic changes that were not captured within this study. Second, our results are susceptible to recall bias as we used patient-reported responses. However, this is unlikely to meaningfully impact our results, as most patients were immobilized for a short period of time consisting of less than 10 weeks. Lastly, all patients had hand or wrist orthotics. Therefore, our results may not be generalizable to orthotics placed on the lower extremity.

## Conclusions

In conclusion, almost a third of patients required at least one unplanned orthotic replacement during their course of immobilization. Despite all patients receiving written and verbal instructions on orthotic care, most patients did not recall receiving instructions. Healthcare providers should be aware of these challenges and aim to improve patient education and retention, especially considering that improper orthotic care and unplanned replacements may place patients at risk for poor outcomes.
